# Analysis of risk factors for acute attacks complicated by respiratory failure in children with asthma

**DOI:** 10.3389/fped.2023.1335540

**Published:** 2024-01-15

**Authors:** Peng Han, Anxia Jiao, Ju Yin, Huimin Zou, Yuliang Liu, Zheng Li, Quan Wang, Jie Wu, Kunling Shen

**Affiliations:** ^1^ Respiratory Department, China National Clinical Research Center of Respiratory Diseases, Beijing, China, Children’s Hospital, Capital Medical University, National Center for Children’s Health, Beijing, China; ^2^ Department of Interventional Pulmonology, Beijing Children’s Hospital, National Center for Children’s Health, Capital Medical University, Beijing, China; ^3^ Pediatric Intensive Care Unit, Beijing Children’s Hospital, Capital Medical University, National Center for Children’s Health, Beijing, China; ^4^ Department of Emergency, Beijing Children’s Hospital, Capital Medical University, National Center for Children’s Health, Beijing, China; ^5^ Department of Pediatrics, Shenzhen Children’s Hospital, Shenzhen, China

**Keywords:** respiratory failure, asthma attack, child, risk factor, hospitalization

## Abstract

**Objective:**

To describe the proportion and clinical characteristics of hospitalized children with acute asthma attacks complicated by respiratory failure and to analyze the risk factors.

**Methods:**

This retrospective study analyzed hospital admissions of children and adolescents with acute asthma attacks between January 2016 and December 2021. Inclusion criteria were used to identify eligible cases, and demographic information and disease characteristics were collected. Patients were categorized into respiratory failure group and the other group based on the result of artery blood gas analysis. Multivariate logistic regression was utilized to investigate the risk factors associated with respiratory failure resulting from acute asthma attacks. The data were analyzed using SPSS 22.0, and significance was considered at *P *< 0.05.

**Results:**

Our research involved 225 participants, with 18.7% diagnosed with respiratory failure. The respiratory failure group was found to be younger and have higher percentage of male, while birth weight, nationality, and type of residence did not differ between the two groups. In the respiratory failure group, a significant difference was observed in emergency hospitalization, ICU treatment, severe to critical attack, dyspnea and allergy history. The two groups did not differ in admission season, first asthma diagnosis, respiratory infection and comorbidity. The respiratory failure group exhibited a higher proportion of atopy-only asthma and a lower proportion of T2-high asthma. The eosinophil count, and eosinophil percentage were lower in the respiratory failure group, while neutrophil count was higher. Having a history of allergies (OR = 2.46, 95% CI: 1.08–5.59) and neutrophil count (OR = 1.10, 95% CI: 1.00–1.21) were the risk factors for respiratory failure in children with asthma. There also existed that the risk of respiratory failure increases with decreasing age of the children (OR = 0.85, 95% CI: 0.73–0.99).

**Conclusion:**

Notably, risk factors for respiratory failure in hospitalized asthma children include age, having a history of allergies, and neutrophil count. The identification of the above factors and the implementation of timely intervention can optimize the treatment of asthma in children.

## 1 Introduction

Respiratory failure is not rare encountered in clinical settings and is considered a serious emergency that often results in admission to pediatric intensive care units (PICU). Due to the heterogeneity of diagnostic criteria, the epidemiology of respiratory failure in children remains unclear. Studies indicated that the prevalence of respiratory distress syndrome in children complicated by respiratory failure was 2.3%, with a mortality rate ranging from 24% to 34% (1, 2). Bronchial smooth muscle spasm is the main pathophysiological characteristics of an acute asthma attack and causes the symptoms such as shortness of breath, dyspnea or wheezing. Excessive saliva-like sputum can also occur during the attack, making it difficult to clear by coughing. Mucus that only partially blocks the airway will gradually thicken into plugs. As the majority of the airway is occupied by the mucus plug, airflow is restricted, leading to more severe respiratory distress, respiratory failure, and even life-threatening conditions (3, 4). The proportion and risk factors of respiratory failure in hospitalized children with acute attacks of asthma were still unclear. Therefore, this study described the proportion and clinical characteristics of hospitalized children with acute asthma attacks complicated by respiratory failure and analyzed the risk factors associated with respiratory failure.

## 2 Method

### 2.1 Subjects

This was a retrospective study. Subjects typically present to the hospital with an acute asthma attack through the outpatient department or the emergency department. The inclusion criteria for the study were children and adolescents who were admission to Beijing Children's Hospital affiliated with Capital Medical University for acute asthma attacks from January 2016 to December 2021. Exclusion criteria included age <3 years old; incomplete demographic information; lack of information on diagnosis and treatment after admission; hospital stay ≤1 day; without arterial blood gas analysis after admission; with underlying diseases such as primary or secondary immunodeficiency disease, hereditary metabolic disease, tumor, and organ or hematopoietic stem cell transplantation; suffering from active pulmonary tuberculosis and with a history of asthma but hospitalized for other reasons.

The electronic case system includes cases diagnosed with bronchial asthma, namely cases coded as J45.0∼J45.9 and J46 in the International Statistical Classification of Diseases and Related Health Problems 10 (ICD-10). The diagnosis of asthma and acute asthma attacks was based on the criteria of “Guidelines for diagnosis and prevention of bronchial asthma in children (2016 Edition)” (5). The diagnostic criteria for asthma were described as: history of typical variable respiratory symptoms (included wheeze, shortness of breath, chest tightness and cough), and confirmed variable expiratory airflow limitation (documented excessive variability in lung function and documented expiratory airflow limitation (5). Diagnostic criteria for respiratory failure were the artery blood gas analysis revealing a PaO2 < 50 mm of mercury (mmHg; 1 mmHg = 0.133 kPa) and/or a PaCO2 > 50 mmHg. PaO2 < 50 mmHg and PaCO2 < 50 mmHg were classified as Type I respiratory failure. PaO2 < 50 mmHg and PaCO2 > 50 mmHg were classified as Type II respiratory failure. The diagnosis of respiratory infection was mainly based on acute onset, fever, expectoration and other respiratory symptoms and signs such as moist rales, combined with whole blood cell analysis, C-reactive protein,, and chest imaging examination, etc.

The included cases were retrieved and information on demographics (such as age, gender, birth weight, etc), pre-admission status (such as allergy history, asthma hospitalization history, history of systemic glucocorticoid therapy, etc), respiratory infection, comorbidities, and disease characteristics (such as asthma phenotype, results of serum allergen detection and whole blood cell analysis, etc) were collected using a pre-designed case report form.

Depending on the presence of respiratory failure, subjects were divided into the respiratory failure group and the other group. The severity classification was also based on the criteria set by the Chinese Guidelines, which classified acute asthma attacks as mild, moderate, severe, or severe for children aged ≥6 years, and mild or severe for children aged <6 years (5). Asthma phenotypes in children were classified as either atopy-only (with blood eosinophils <470/μl, the sum of any sIgE ≥0.7 ku/l), Eos-only (with blood eosinophils ≥470/μl and all sIgE <0. 7 ku/l), T2-high (with blood eosinophils ≥470/μl and any sIgE ≥0.7 kU/l), or T2-low (with blood eosinophils <470/μl and all sIgE <0.7 ku/l) (6, 7). The choice for performing a bronchoscopy examination was based on the physician's professional opinion after clinical consultation and physical examination. It is our routine practice to perform a bronchoscopy typically once the acute attack has resolved and before discharge and we will present the findings from this.

This trial protocol was approved by the Ethics Committee of Beijing Children's Hospital affiliated with Capital Medical University on July 13, 2023 [the ethical approval number: (2023)-E-078-Y]. The study was registered at https://clinicaltrials.gov/ with the number: NCT05800379.

### 2.2 Statistical analysis

The normality of continuous variables was verified through the Kolmogorov–Smirnov test. For normally distributed continuous measurement data, mean and standard deviation was used to express it, and for group comparisons, independent sample *t*-tests were conducted. In contrast, median and quartile intervals were used to express non-normally distributed measurement data, and group comparisons were made using the Mann–Whitney-U rank sum test. The frequency and percentage (%) of counting data were described and compared between groups using a chi-square test. Multiple comparisons between groups were conducted using the Bonferroni correction. Multivariate logistic regression was utilized to examine the risk factors associated with respiratory failure with acute asthma attacks. The variables with a *p*-value of ≤0.20 were chosen as candidates for multivariate logistic regression analysis. The Hosmer–Lemeshow test assessed the degree of model fitting. The data were analyzed using SPSS 22.0, and significance was considered at *P *< 0.05.

## 3 Result

### 3.1 General information on the enrolled subjects

**Figure 1:**
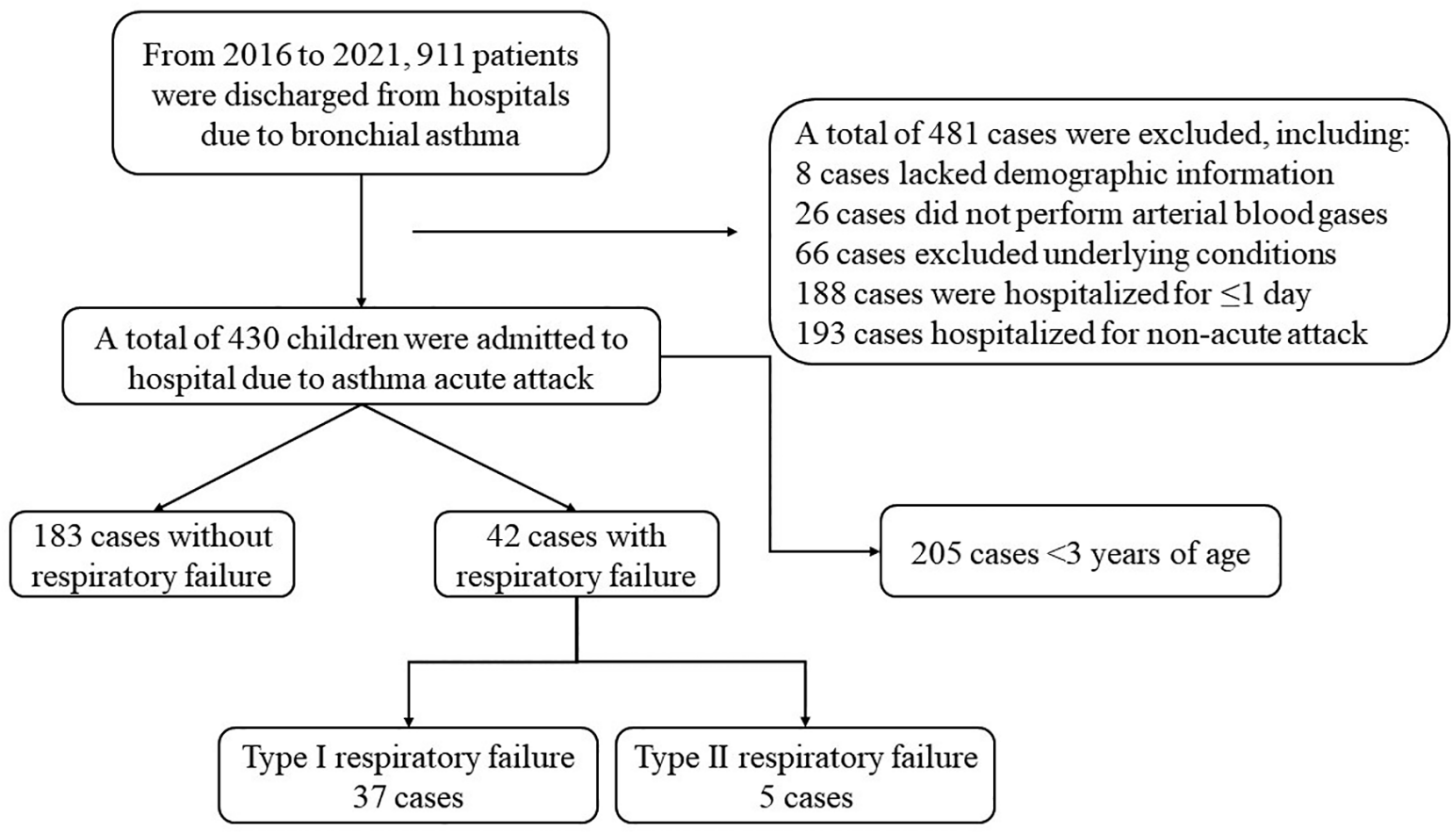
Flowchart of enrolment and exclusion of subjects.

In the electronic medical record system, 911 cases that met the inclusion criteria were retrieved. A total of 225 subjects participated in this study, as illustrated in Figure 1. Among them, 42 (18.7%) presented with respiratory failure, while 183 (81.3%) did not. Out of the 42 children with respiratory failure, 37 children were diagnosed with type I and 5 children with type II. The participants consisted of 132 male children (58.7%) and 93 female children (41.3%) with the age range of 3.00–17.08 years.

### 3.2 Comparison of clinical data of subjects in two groups

In our study, the age of children with respiratory failure was lower (4.42 years vs. 6.75 years, *P* = 0.001); and the percentage of male in respiratory failure group was higher (73.8% vs. 55.2%, *P* = 0.027). Table 1 revealed that there were no significant variations in nationality, and type of residence between the respiratory failure group and the other group (*P* > 0.05).

**Table 1:** Comparison of general characteristics of subjects.

	Respiratory failure group (*N* = 42)	The other group (*N* = 183)	Z/*χ*^2^	*P*-value
Age (years)	4.42 (3.50–7.42)*	6.75 (4.67–9.25)	−3.334^a^	0.00
Male	31 (73.8%)*	101 (55.2%)	4.883	0.03
Han ethnic	37 (88.1%)	171 (93.4%)	1.398	0.24
Urban living	34 (81.0%)	140 (76.5%)	0.386	0.53
Birth weight (kg)	3.30 (3.00–3.70)	3.30 (3.00–3.60)	−0.003^a^	0.99
Premature	2 (4.9%)	12 (6.56%)	0.627	0.73
Cesarean	20 (47.6%)	87 (47.8%)	0.063	0.80
Neonatal asphyxia	0 (0.00%)	1 (0.6%)	1.508	0.47
Neonatal oxygen	0 (0.00%)	4 (2.2%)	2.206	0.33
Feeding mode			1.989	0.37
Breastfeeding	28 (66.7%)	127 (69.4%)		
Formula feeding	3 (7.1%)	25 (13.7%)		
Mixed feeding	9 (21.4%)	28 (15.3%)		
Allergy history	20 (47.6%)*	69 (37.7%)	6.067	0.05
Allergic family history	19 (45.2%)	84 (46.2%)	0.019	0.89
Siblings ≥ 1	19 (45.2%)	72 (39.3%)	0.493	0.48
Asthma hospitalization history	6 (14.3%)	32 (17.5%)	0.249	0.62
History of systemic glucocorticoid therapy	6 (14.3%)	15(8.2%)	1.717	0.19

^a^ The Mann–Whitney *U*-test was used for group comparisons.

* *P *< 0.05.

Compared to subjects without respiratory failure, our study found the respiratory failure group had a higher percentage with a positive allergy history (47.6% vs. 37.7%, *P *= 0.048). As Table 1 showed, there was no significant disparity between the two groups in terms of birth weight, premature infants, delivery mode, neonatal asphyxia, neonatal oxygen inhalation, feeding mode, allergic family history, asthma hospitalization history, and systemic glucocorticoid therapy (*P* > 0.05).

Our study indicated that the respiratory failure group had a significantly higher proportion of emergency attendance (97.6% vs. 53.0%, *P *< 0.001) and PICU treatment (76.2% vs. 9.3%, *P *< 0.001). The proportion of atopy-only asthma was higher in subjects with respiratory failure (70.7% vs. 61.3%). While the proportion of T2-high asthma was lower (2.4% vs. 22.0%). There was no difference in terms of admission season, first asthma diagnosis, respiratory infection and comorbidity between the two groups, as shown in Table 2.

**Table 2:** Comparison of the characteristics of subjects after admission.

	Respiratory failure group (*N* = 42)	The other group (*N* = 183)	Z/χ^2^	*P-*value
Admission season			3.829	0.28
Spring	3 (7.1%)	35 (19.1%)		
Summer	9 (21.4%)	40 (21.9%)		
Autumn	23 (54.8%)	85 (46.5%)		
Winter	7 (16.7%)	23 (15.6%)		
Emergency admission	41 (97.6%)*	97 (53.0%)	28.669	0.00
PICU treatment	32 (76.2%)*	17 (9.3%)	89.751	0.00
First asthma diagnoses	35 (83.3%)	155 (84.7%)	0.049	0.83
Respiratory infection	39 (92.9%)	158 (86.34%)	1.332	0.25
Comorbidity	23 (54.8%)	95 (51.9%)	0.111	0.79
Asthma phenotype			9.167	0.03
Atopy-only	29 (70.7%)*	92 (61.3%)		
Eos-only	2 (4.9%)	4 (2.7%)		
T2-high	1 (2.4%)*	33 (22.0%)		
T2-low	9 (22.0%)	21 (14.0%)		
Severe or critical attacks	26 (61.9%)*	50 (27.3%)	18.264	0.00
Dyspnea	30 (71.4%)*	79 (43.2%)	10.922	0.00
Mucus plugging of Bronchoscopy examination^a^	13 (59.1%)	78 (65.0%)	0.315	0.85
Positive serum allergen detection	31 (73.8%)*	139 (76.0%)	0.085	0.77
Procalcitonin	0.17 (0.09–0.36)	0.11 (0.06–0.38)	−1.175	0.24
C-reactive protein	9.00 (4.00–16.00)	8.00 (3.00–15.70)	−0.875	0.38
White blood cell counts	9.11 (7.57–13.22)	9.14 (6.99–12.02)	0.133	0.72
Neutrophil count	6.99 (4.62–10.90)*	5.82 (3.40–8.47)	−1.958	0.05
Eosinophil count	0.02 (0.01–0.08)*	0.13 (0.02–0.36)	−8.389	0.00
Eosinophil percentage	0.20 (0.10–0.80)*	1.25 (0.20–3.90)	−3.415	0.00
Abnormal chest CT findings	36(92.3%)	144(88.3%)	0.183	0.67

^a^ A total of 142 children underwent bronchoscopy, including 22 cases in respiratory failure group and 120 cases in the other group.

* *P *< 0.05.

In our study, the respiratory failure group demonstrated a lower eosinophil percentage (median: 0.20% vs. 1.25%, *P* = 0.001) and eosinophil count (median: 0.02*10^9/l vs. 0.13*10^9/l, *P *< 0.001). In addition, neutrophil count was increased in the respiratory failure group (median: 6.99*10^9/l vs. 5.82*10^9/l, *P *= 0.05). As shown in Table 2, there were no statistical differences in the formation of mucus plugs under bronchoscopy, procalcitonin, C-reactive protein, white blood cell counts, or proportion of abnormal chest CT findings between the two groups (*P* > 0.05).

### 3.3 Multivariate logistic regression analysis of risk factors in subjects with respiratory failure

After comparing the general data and clinical features of the two groups, we identified variables with statistically significant differences (*P* < 0.20), which were used as independent variables. We assigned the presence or absence of respiratory failure as the dependent variable, denoting absence as 0 and presence as 1.

The independent variables comprise eight items: age, gender, allergic history, history of systemic glucocorticoid therapy, asthma phenotype, neutrophil count, eosinophil count, and the percentage of eosinophils. The assignment of variables was displayed in Table 3.

**Table 3:** Variable assignment in regression analysis of subjects with respiratory failure.

Variable name	Assignment			
Gender	0 = Male		1 = Female	
Allergy history	0 = Without		1 = With	
History of systemic glucocorticoid therapy	0 = Without		1 = With	
Asthma phenotype	0 = Atopy-only	1 = Eos-only	2 = T2-high	3 = T2-low

The logistic regression model contained the aforementioned eight variables, with an introduction level of 0.05 and an exclusion level of 0.10. Multivariate logistic regression was performed to analyze the risk factors for respiratory failure in children with asthma. As shown in Table 4, having a history of allergies (OR = 2.46, 95% CI: 1.08–5.59) and neutrophil count (OR = 1.10, 95% CI: 1.00–1.21) were the risk factors for respiratory failure in children with asthma. There was a negative correlation between age and respiratory failure in asthma children (OR = 0.85, 95% CI: 0.73–0.99), whereby younger asthma children exhibited a higher risk of respiratory failure.

**Table 4:** Results of multivariable logistic regression analysis.

Risk factors	*β*	*P*-value	OR value	95% CI
Constant	1.314	0.24	3.72	
Age	−0.158	0.05	0.85	0.73–0.99
Male	−0.844	0.06	0.43	0.18–1.02
Neutrophil count	0.096	0.05	1.10	1.00–1.21
Having a history of allergies	0.901	0.03	2.46	1.08–5.59
Eosinophil count	−5.736	0.08	0.03	0.00–1.79
Percentage of eosinophils	0.141	0.61	1.15	0.67–1.97
History of systemic glucocorticoid therapy	−0.762	0.28	0.47	0.12–1.83
Asthma phenotype (relative to T2 low group)				
Atopy-only	−0.445	0.38	0.64	0.24–1.75
Eos-only	2.909	0.08	18.34	0.72–470.91
T2-high	−0.712	0.56	0.49	0.05–5.25

CI, Confidence interval.

Hosmer–Lemeshow test: Chi-square = 4.132, significance = 0.846, and the model fitted well.

## 4 Discussion

Respiratory failure is a frequent and critical emergency in the clinic. It is one of the most common reasons for admission to the PICU. The main pathophysiological mechanism of respiratory failure is that lung ventilation and/or ventilation dysfunction cannot effectively exchange oxygen. This can result in clinical syndromes including aerobic metabolism disorder, insufficient energy production, cell damage, and dysfunction requiring non-invasive respiratory support and even mechanical ventilation treatment (8). Respiratory failure can be caused by various reasons and be classified into the following three categories: lung parenchymal disease, airway obstruction, or neuromuscular dysfunction. When it arises from lung diseases, respiratory failure results from an imbalance in the ventilation-blood flow ratio, a disorder in gas diffusion, or both.

Our study investigated the proportion of respiratory failure and associated risk factors in children with asthma who were admitted to the hospital. In our study, it was found that hospitalized children experiencing acute asthma attacks led to a respiratory failure rate of 18.7%. All subjects in our study were treated in a tertiary hospital which was also a national children's medical center; this reduces the applicability of the results to a wider audience. In the analysis process, 26 cases were excluded because of not performing arterial blood gases. Though there may be a bias of disease severity in children with asthma which could cause the proportion of respiratory failure higher than general asthma children, this proportion represented the situation in our hospital.

In order to better distinguish the effects of asthma and respiratory infection on respiratory failure, we excluded children under 3 years old who may more likely have had bronchiolitis rather than a true asthma attack in the study enrollment stage. The univariate analysis revealed no disparity in the frequency of respiratory tract infections among the two groups. So, in the process of analysis, the influence of respiratory tract infection on respiratory failure were similar. Respiratory tract infection is one of the causes of respiratory failure and also a common factor that contributes to acute asthma attacks which might be a concomitant factor that should not be overlooked (9, 10).

Children with a smaller airway radius are more susceptible to respiratory failure compared to adults. The increased airflow resistance stems from the narrower diameter of their airways. Resistance increases inversely with the fourth power of the airway radius (11). A minor change in the diameter of the airway can significantly increase resistance and drastically reduce airflow. This is consistent with our finding that the younger the hospitalized child with asthma, the higher the risk of respiratory failure. The contraction of airway smooth muscle and the accumulation of mucus in the airways contribute to respiratory failure, which could result in fatal asthma. In children, the airway is further narrowed by secretions or mucus plugs, and slight contractions of airway smooth muscle substantially increase resistance to gas flow (12). Fatal asthma attacks display a bimodal distribution based on duration. Short course of time to death from the onset of the fatal attack result from highly contraction of airway smooth muscle, whereas long course of time occurs due to obstruction of the airways due to mucus (13). Fatal asthma features histopathologic characteristics including airway remodeling, inflammatory cell infiltration, mucus plug obstruction, and smooth muscle contraction (14, 15).

According to Maggi JC et al., children with asthma who underwent tracheal intubation and mechanical ventilation, and experienced respiratory failure, all showed the presence of mucus plugs or plastic formations during bronchoscopy (16). Nevertheless, our study showed that only 59.1% of subjects in the respiratory failure group were able to visualize mucus plugs during bronchoscopy examination. The discrepancy primarily stems from the circumstance that the prior study only examined a restricted sample of subjects requiring tracheal intubation in the PICU. In contrast, our study also concentrated on children with asthma hospitalized on the general ward for therapy.

The severity of acute attacks and dyspnea in hospitalized children were relative factors for respiratory failure in the study which are not particularly helpful in a clinical setting. Besides dyspnea in itself is a symptom of respiratory failure. So, in the subsequent multivariable logistic regression analysis we did not include these two factors.

Our study had some limitations. First, this study was a retrospective study which may combined with a sampling bias. Given the grade of the hospital, there may be a bias of disease severity in children with asthma who were hospitalized for treatment. This could result in a higher proportion of respiratory failure among children with asthma who need hospitalized. Additionally, retrospective research methods resulted in incomplete or unavailable clinical information and laboratory test results for individual children. A total of 142 children underwent bronchoscopy examination which only account 63.1% of study subjects. Therefore, it difficult to clarify the relationship between mucus plugs and respiratory failure in asthma children. While it is not safe to perform routine bronchoscopy during the peak of an acute asthma attack, we performed brochoscopy when the acute attack was resolving and we therefore may have missed the acute findings. This is also the content that we will study in the near future. Finally, some common objective indicators before enrollment (ACT, c-ACT, etc.) and treatment before hospitalized in subjects were unattainable. Glucocorticoid therapy has significant effects on eosinophil count and percentage. No association between eosinophils and respiratory failure were found in this study, which needs to be further explored in multi-center prospective clinical research. And this might also influence the result of asthma phenotype, but classifying asthma phenotype using serum IgE and eosinophils counts was a significant endeavor. Nevertheless, the widespread adoption of electronic medical records has made observational research, which can provide clinical insights, more feasible. Clinical data obtained from electronic medical records can facilitate the effective utilization of available data. Our research was conducted using a clinic-based data source, which ensured the reliability of our findings.

## 5 Conclusion

Respiratory failure in hospitalized asthma children was associated with various factors, including age, gender, asthma phenotype, allergy history, severity, history of systemic glucocorticoid therapy, neutrophil count, eosinophil count, and eosinophil percentage. Notably, relative factors for respiratory failure include age, having a history of allergies, and neutrophil count detection. The identification of the above relative factors and the implementation of timely intervention can optimize the treatment of asthma in children.

## Data Availability

The original contributions presented in the study are included in the article/Supplementary Material, further inquiries can be directed to the corresponding author.
